# Supplementary material for: FERN – a Java framework for stochastic simulation and evaluation of reaction networks
Source: BMC Bioinformatics. 2008 Aug 29;9:356. doi: 10.1186/1471-2105-9-356 (PMC2553347; doi:10.1186/1471-2105-9-356)
Supplement: Additional file 1 — FERN distribution, Version 1.3. This archive contains the FERN source code and binaries as well as documentation and example models in FernML and SBML. [file 1471-2105-9-356-S1.zip › fern/doc/javadoc/fern/simulation/algorithm/TauLeapingSpeciesPopulationBoundSimulator.html]

TauLeapingSpeciesPopulationBoundSimulator


---


|  |  |  |
| --- | --- | --- |
| **Overview** \| **Package** \| **Class** \| **Use** \| **Tree** \| **Deprecated** \| **Index** \| **Help** | |  |
| **PREV CLASS**   NEXT CLASS | **FRAMES**    **NO FRAMES**     **All Classes** |  |
| SUMMARY: NESTED \| FIELD \| CONSTR \| METHOD | DETAIL: FIELD \| CONSTR \| METHOD |  |


---


## fern.simulation.algorithm Class TauLeapingSpeciesPopulationBoundSimulator

```
java.lang.Object
  fern.simulation.Simulator
      fern.simulation.algorithm.GillespieEnhanced
          fern.simulation.algorithm.AbstractBaseTauLeaping
              fern.simulation.algorithm.TauLeapingSpeciesPopulationBoundSimulator
```

---

``` public class TauLeapingSpeciesPopulationBoundSimulator extends AbstractBaseTauLeaping ```

There are some possibilities to bind the expected change of the propensities by a
value epsilon in order to fulfill the leap condition. Here it is bound indirectly
by the reactant species of each reaction which gives the same leaps in a more
efficient manner.

Daniel T. Gillespie, Approximate accelerated stochastic simulation
of chemically reacting systems, Journal of chemical physics vol 115, nr 4 (2001); Cao et al., Efficient
step size selection for the tau-leaping simulation method, Journal of chemical physics 124, 044109 (2006)

**Author:**
:   Florian Erhard

---

**Nested Class Summary**

**Nested classes/interfaces inherited from class fern.simulation.Simulator**

`Simulator.FireType`


**Field Summary**

**Fields inherited from class fern.simulation.algorithm.AbstractBaseTauLeaping**

`productHistos, reactantHistos, verbose`

**Fields inherited from class fern.simulation.algorithm.GillespieEnhanced**

`a_sum`

**Fields inherited from class fern.simulation.Simulator**

`a, stochastics, t`


**Constructor Summary**

`TauLeapingSpeciesPopulationBoundSimulator(Network net)`


| **Method Summary** | |
| --- | --- |
| `protected  double` | `chooseTauNonCriticals(BitVector criticals)`             choose the timestep candidate for the noncritical reactions by some method. |
| `String` | `getName()`             Returns the name of this simulator |

**Methods inherited from class fern.simulation.algorithm.AbstractBaseTauLeaping**

`chooseTauCriticals, getEpsilon, getLangevinThreshold, getNCritical, getNumSimpleCalls, getUseSimpleFactor, getV, isVerbose, performStep, setEpsilon, setLangevinThreshold, setNCritical, setNumSimpleCalls, setUseSimpleFactor, setVerbose`

**Methods inherited from class fern.simulation.algorithm.GillespieEnhanced**

`directMCTau, initialize, isEfficientlyAdaptSum, reinitialize, setAmount, setEfficientlyAdaptSum, setVolume`

**Methods inherited from class fern.simulation.Simulator**

`addObserver, fireReaction, fireReaction, getAmount, getAmountManager, getNet, getNextThetaEvent, getPropensity, getPropensityCalculator, getTime, getVolume, isInterpolateTheta, registerNewTheta, setInterpolateTheta, start, start, thetaEvent`

**Methods inherited from class java.lang.Object**

`clone, equals, finalize, getClass, hashCode, notify, notifyAll, toString, wait, wait, wait`

**Constructor Detail**

### TauLeapingSpeciesPopulationBoundSimulator

```
public TauLeapingSpeciesPopulationBoundSimulator(Network net)
```


**Method Detail**

### chooseTauNonCriticals

```
protected double chooseTauNonCriticals(BitVector criticals)
```

:   **Description copied from class: `AbstractBaseTauLeaping`**
:   choose the timestep candidate for the noncritical reactions by some method.

    :   **Specified by:**: `chooseTauNonCriticals` in class `AbstractBaseTauLeaping`
    :   **Parameters:**: `criticals` - Bitvector identifying the critical reactions **Returns:**: timestep candidate for noncritical reactions

---


### getName

```
public String getName()
```

:   **Description copied from class: `AbstractBaseTauLeaping`**
:   Returns the name of this simulator

    :   **Overrides:**: `getName` in class `AbstractBaseTauLeaping`
    :   **Returns:**: simulator name


---


|  |  |  |
| --- | --- | --- |
| **Overview** \| **Package** \| **Class** \| **Use** \| **Tree** \| **Deprecated** \| **Index** \| **Help** | |  |
| **PREV CLASS**   NEXT CLASS | **FRAMES**    **NO FRAMES**     **All Classes** |  |
| SUMMARY: NESTED \| FIELD \| CONSTR \| METHOD | DETAIL: FIELD \| CONSTR \| METHOD |  |


---
